# Hypoxia-Induced Centrosome Amplification Underlies Aggressive Disease Course in HPV-Negative Oropharyngeal Squamous Cell Carcinomas

**DOI:** 10.3390/cancers12020517

**Published:** 2020-02-24

**Authors:** Karuna Mittal, Da Hoon Choi, Guanhao Wei, Jaspreet Kaur, Sergey Klimov, Komal Arora, Christopher C. Griffith, Mukesh Kumar, Precious Imhansi-Jacob, Brian D. Melton, Sonal Bhimji-Pattni, Remus M. Osan, Padmashree Rida, Paweł Golusinski, Ritu Aneja

**Affiliations:** 1 Department of Biology, Georgia State University, Atlanta, GA 30303, USA; kkaruna.goel@gmail.com (K.M.); dchoi@msm.edu (D.H.C.); gwei1@student.gsu.edu (G.W.); jkaur2@student.gsu.edu (J.K.); sklimov1@student.gsu.edu (S.K.); karora@gsu.edu (K.A.); mkumar8@gsu.edu (M.K.); pimhansijacob1@student.gsu.edu (P.I.-J.); meltonb@janelia.hhmi.org (B.D.M.); rosan@gsu.edu (R.M.O.); 2 Emory Hospital Midtown, Atlanta, GA 30308, USA; griffic8@ccf.org (C.C.G.); sonal.pattni1@gmail.com (S.B.-P.); 3 Novazoi Theranostics, Rancho Palos Verdes, CA 90725, USA; cgp_rida@yahoo.com; 4 Department of Otolaryngology and Maxillofacial Surgery, University of Zielona Gora, 65-417 Zielona Gora, Poland; pgolusinski@uz.zgora.pl; 5 Department of Biology and Environmental Sciences, Poznan University of Medical Sciences, 61-701 Poznan, Poland

**Keywords:** hypoxia, human papillomavirus, oropharyngeal squamous cell carcinoma, centrosome amplification, miRNA

## Abstract

Human papillomavirus-negative (HPV-neg) oropharyngeal squamous cell carcinomas (OPSCCs) are associated with poorer overall survival (OS) compared with HPV-positive (HPV-pos) OPSCCs. The major obstacle in improving outcomes of HPV-neg patients is the lack of robust biomarkers and therapeutic targets. Herein, we investigated the role of centrosome amplification (CA) as a prognostic biomarker in HPV-neg OPSCCs. A quantitative evaluation of CA in clinical specimens of OPSCC revealed that (a) HPV-neg OPSCCs exhibit higher CA compared with HPV-pos OPSCCs, and (b) CA was associated with poor OS, even after adjusting for potentially confounding clinicopathologic variables. Contrastingly, CA was higher in HPV-pos cultured cell lines compared to HPV-neg ones. This divergence in CA phenotypes between clinical specimens and cultured cells can therefore be attributed to an inaccurate recapitulation of the in vivo tumor microenvironment in the cultured cell lines, namely a hypoxic environment. The exposure of HPV-neg OPSCC cultured cells to hypoxia or stabilizing HIF-1α genetically increased CA. Both the 26-gene hypoxia signature as well as the overexpression of HIF-1α positively correlated with increased CA in HPV-neg OPSCCs. In addition, we showed that HIF-1α upregulation is associated with the downregulation of miR-34a, increase in CA and expression of cyclin- D1. Our findings demonstrate that the evaluation of CA may aid in therapeutic decision-making, and CA can serve as a promising therapeutic target for HPV-neg OPSCC patients.

## 1. Introduction

Head and neck squamous cell carcinoma (HNSCC) is the sixth most common cancer worldwide [1]. Although the incidence and mortality rates for HNSCC are declining globally, there has been a gradual increase in the incidence rate of oropharyngeal squamous cell carcinomas (OPSCCs) in recent years [2]. OPSCC is a type of HNSCC, which includes cancer of the tonsils, base of the tongue, back of the roof of the mouth and the side and back walls of the throat. A major factor underlying the increased incidence rate of OPSCCs is human papillomavirus (HPV) infection [3]. Studies have shown that HPV-positive (HPV-pos) OPSCC patients respond better to treatment compared to patients with HPV-negative (HPV-neg) OPSCC. A randomized trial comparing accelerated-fractionation with standard-fractionation radiotherapy, in combination with cisplatin therapy showed better rates of three-year overall survival (82.4%) in HPV-pos OPSCC patients compared to their HPV-neg counterparts (57.1%) [4]. In the absence of a good therapeutic target, conventional chemotherapy is unfortunately still the mainstay of treatment for HPV-neg OPSCC patients, whose prognosis remains dismal.

Treatment decisions for OPSCC patients are made based on disease stage and tumor location, without taking into account the tumor’s HPV status [5]. Recent studies have suggested that HPV-pos and HPV-neg OPSCCs are biologically unique entities [4], with different tumor biologies and characteristics. Thus, a new stage categorization has been introduced for HPV-pos OPSCC in the 8th edition TNM [T describes the size of the tumor and any spread of cancer into nearby tissue; N describes spread of cancer to nearby lymph nodes; and M describes metastasis (spread of cancer to other parts of the body)] classification system [6], wherein p16 staining serves as a surrogate for HPV status. Studies have indicated that HNSCC is a heterogeneous disease with higher chromosomal instability (CIN) in HPV-neg (50% higher mutational load) compared to HPV-pos HNSCCs [7]. The inactivation of tumor suppressor genes such as TP53 and CDKNA2 and the oncogenic activation of the *CCND1* gene have been shown to be crucial for pathogenesis and disease progression in HPV-neg HNSCCs [8]. By contrast, the inactivation of the tumor suppressor genes and activation of oncoproteins in HPV-pos tumors have been linked to the viral E6 and E7 oncoproteins [9]. A key feature of E6 and E7 oncoproteins is that they both converge to induce centrosome amplification (CA) [10], a hallmark of cancer. Amplified centrosomes are well-recognized to drive CIN, which fuels tumorigenesis, tumor progression, drug resistance and, as a result, poor prognosis [11]. CA can be numerical (increase in the number of centrosomes) as well as structural (increase in size/volume of centrosomes) and can arise in multiple ways, including the failure of the cell to undergo cytokinesis, inappropriate replication of centrosomes, and de novo generation of centrosomes [12,13].

Past genomic analyses of HNSCCs have described CIN to be a more prominent feature in HPV-neg tumors than in HPV-pos tumors [14]. Drivers of CA are distinct in HPV-pos and HPV-neg HNSCCs; CA in the former subtype is driven by viral oncoproteins, and that in the latter is driven by the misexpression of host cell-encoded proteins. Aurora A kinase and PLK1 are major factors contributing to CIN in HPV-neg HNSCCs [15]. Both Aurora A and PLK1 promote CIN by deregulating the spindle assembly checkpoint, resulting in chromosome mis-segregation and the amplification of centrosomes [16]. Therefore, we reasoned that CA may be a readily quantifiable prognostic biomarker and a potentially druggable target for HPV-neg tumors.

Another critical factor underlying poorer prognosis in patients with HPV-neg cancers is tumor hypoxia. Tumor hypoxia has long been associated with poor responses to radiotherapy and chemotherapy [17]. A recent study has shown that HPV-neg oropharyngeal tumors display higher tumor hypoxia [18]. Additionally, reduced partial pressure of oxygen inside tumors plays a significant role in overexpression of Aurora-A/STK15 [19], and this overexpression results in CA, CIN, and aneuploidy. The hypoxia-mediated overexpression of PLK4 has been well-documented to induce CA [20]. Recently, we have shown that hypoxic tumor microenvironment can induce CA via the stabilization of the transcriptional factor HIF-1α in breast cancer, facilitating an aggressive disease course [21]. Thus, there is mounting evidence that hypoxia-associated CA may underlie the aggressive disease course and treatment resistance of HPV-neg OPSCCs.

No studies to date have reported quantitation of centrosomal aberrations in OPSCCs with inherently different HPV status. Herein, we performed a thorough quantitative analysis of centrosomal aberrations in OPSCC tumors to compare differences in incidence and severity of CA between HPV-pos and HPV-neg OPSCCs. Interestingly, we found that HPV-neg OPSCCs exhibited significantly higher CA when compared with HPV-pos OPSCCs, and CA was associated with the poor overall survival in HPV-neg OPSCCs. Furthermore, we found that HPV-neg tumors show a higher expression of HIF-1α than HPV-pos OPSCCs, and there was a strong association between CA and HIF-1α expression in HPV-neg OPSCCs. In addition, we found that HPV-neg tumors exhibited a higher expression of the CA-associated protein, cyclin D1. Our study also showed that HIF-1α upregulation is associated with the downregulation of miR-34a, an increase in CA and the expression of cyclin- D1. Taken together, these results shed new light on the drivers of tumor biology in HPV-neg tumors and emphasize the role of CA as new prognostic marker and actionable target to improve outcomes in HPV-neg OPSCC patients.

## 2. Results

### 2.1. Discordant Relationship between CA in Cultured Cells versus Patient Samples for HPV-Pos and HPV-Neg OPSCC

**Figure 1:**
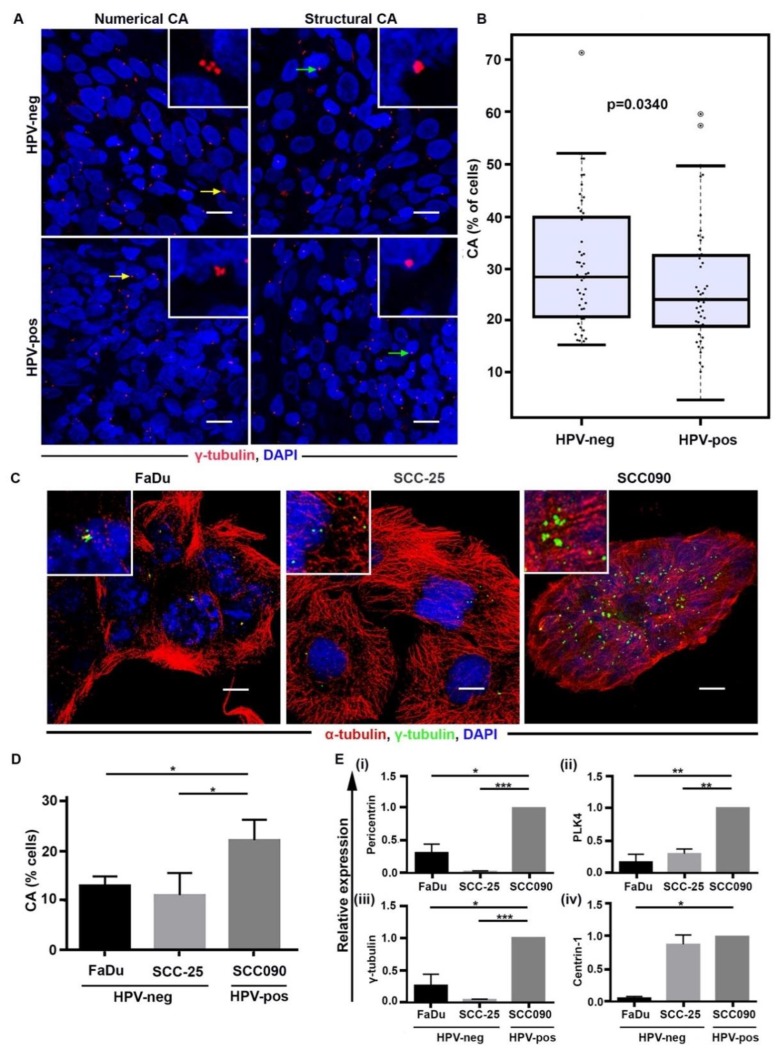
HPV-neg OPSCC specimens exhibit higher CA when compared with the HPV-pos OPSCCs. (**A**) Confocal immunomicrographs showing numerical and structural CA in HPV-pos and HPV-neg OPSCC sections. OPSCC tissue sections were immunostained for centrosomes (γ-tubulin, red) and counterstained with Hoechst (blue). Scale bar (white), 20 μm. (**B**) Percentage distribution of cells with CA (structural and numerical) in HPV-neg (n = 51) and HPV-pos (n = 47) patients (*p* < 0.0340). (**C**) Confocal immunomicrographs showing numerical CA in HPV-pos and HPV-neg tumor cells. OPSCC cells were immunostained for centrosomes (γ-tubulin, green), microtubules (α- tubulin, red) and counterstained with Hoechst (blue). Scale bar (white), 20 μm. (**D**) Percentage distribution of cells with CA (structural and numerical) in HPV-neg and HPV-pos cells (*p* < 0.05). (**E**) qRT-PCR analysis of mRNAs for γ-tubulin, pericentrin, centrin-1, and PLK4 in FaDu, SCC25, and SCC090 cells. Data were normalized by the amount of GAPDH mRNA, expressed relative to the corresponding value for all the cells and are means ± SD from triplicate data. * *p* ≤ 0.05, ** *p* ≤ 0.01, *** *p* ≤ 0.001, **** *p* ≤ 0.0001.

Previous studies from our group have shown that higher levels of CA are associated with poor prognosis and aggressive disease course in multiple malignancies, including breast cancer, pancreatic cancer, and serous ovarian adenocarcinoma [22,23,24]. We are the first to perform a rigorous quantitative study to compare CA in HPV-pos and HPV-neg OPSCCs. Given that the HPV-neg OPSCCs exhibit a higher expression of DNA damage response genes and are associated with a more aggressive disease course and poorer overall survival than HPV-pos OPSCCs, we postulated that HPV-neg tumors might exhibit significant CA. In this study, centrosomes in resection samples from 98 OPSCC patients (n = 47 HPV-pos and n = 51 HPV-neg samples) were visualized by immunofluorescence confocal imaging (details in supplementary information). Centrosome numbers and volumes were evaluated to generate a cumulative percentage of structurally and numerically amplified centrosomes for each sample (patient cohort details are shown in Table 1). Wilcoxon distribution scores revealed that HPV-neg OPSCCs exhibit significantly higher CA (numerical and structural) compared to HPV-pos OPSCCs (*p* = 0.034; Figure 1A,B). In agreement with the view that CA drives tumor progression, our data (Figure S1A) showed that, among HPV-neg tumors, higher CA was associated with a higher disease stage (*p* = 0.1013). Moreover, HPV-neg tumors displayed higher CA compared to stage-matched (stage III and stage IV; *p* = 0.1225 and *p* = 0.0551, respectively) HPV-pos tumors (Figure S1B,C).

However, when cultured cells from HPV-pos and HPV-neg OPSCCs were stained for CA, we observed that HPV-pos OPSCC cells (SCC090) exhibit higher CA (*p* < 0.05) relative to the HPV-neg OPSCC cells (FaDu and SCC-25) (Figure 1C,D). Our findings from RT-PCR also indicate significantly higher levels of centrosomal (pericentrin, γ-tubulin and centrin-1) and CA-associated (PLK4) mRNA expression in HPV-pos OPSCC cells compared to levels observed in HPV-neg OPSCC cells (Figure 1E). Taken together, these findings clearly demonstrate a high prevalence of CA in HPV-neg OPSCC tissue sections from patient samples, but not in cultured cell lines, and suggest that differences between the in vivo tumor microenvironment and in vitro culture conditions are at least partly responsible for this discrepant observation.

### 2.2. CA Is Associated with Poor Overall Survival in HPV-Neg OPSCCs

**Figure 2:**
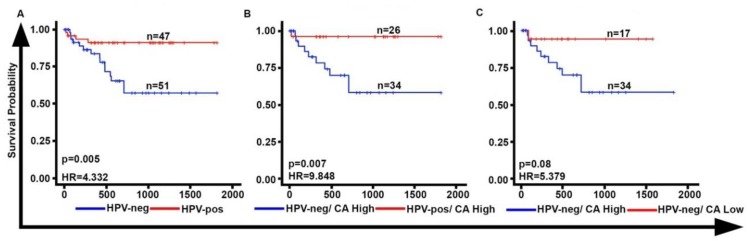
CA is associated with poor overall survival in HPV-neg OPSCCs. (**A**) Kaplan Meier survival curves representing the survival probabilities of HPV-neg (n = 51) and HPV-pos (n = 47) OPSCC patients (HR = 4.629; *p* = 0.0062), (**B**) Kaplan Meier survival curves representing the survival probabilities of high-CA HPV-neg (n = 34) and HPV-pos (n = 26) OPSCC patients (HR = 5.07; *p* = 0.003), and (**C**) Kaplan Meier survival curves representing the survival probabilities of high-CA HPV-neg (n = 34) and low CA HPV-neg (n = 17) OPSCC patients (HR = 5.379, *p* = 0.08).

Consistent with previous studies, our data show that patients with HPV-neg OPSCCs had poorer overall survival when compared to patients with HPV-pos OPSCCs (HR = 4.332; *p* = 0.005) (Figure 2A). When HPV-pos and HPV-neg patients were stratified into low- and high-CA (threshold used was the one that minimized log-rank *p*-value), we found that high-CA HPV-neg OPSCCs were associated with a poorer overall survival relative to high-CA HPV-pos OPSCCs (HR = 9.848; *p* = 0.007) (Figure 2B). Furthermore, within HPV-neg OPSCCs, the high-CA subgroup was associated with worse overall survival relative to the subgroup with low-CA HPV-neg OPSCCs (Figure 2C). This association of overall survival with CA was significant (HR = 7.3; *p* = 0.02) in our multivariable analysis when potentially confounding factors like smoking, alcohol consumption, grade and tumor stage were accounted for (Table 2). In univariate and multivariate analyses, only CA remained significantly associated with OS (Table 2). In contrast, no significant differences were found between the overall survival of low-CA HPV-pos and HPV-neg subgroups. Taken together, HPV-neg OPSCCs exhibit higher CA when compared with HPV-pos OPSCCs and higher CA in the HPV-neg OPSCCs is associated with poorer overall survival.

To strengthen our clinical findings, we performed an in silico analysis of the publicly available the cancer genome atlas (TCGA) microarray data of HNSCC patients (Table S1) [25]. Herein, we evaluated the expression levels of seven CA-related genes and the associations between this signature and overall survival in these patients. A cumulative score (CA7) was generated by adding the log-transformed values, normalized gene expression for CCND1, NEK2, PIN1, TUBG1, PLK1, BIRC5 and AURKA. First, we evaluated the CA7 score in all (n = 521) HNSCCs, regardless of subtype and HPV status. The patients were then stratified into high- and low-CA subgroups using the optimal CA7 score cut-off point (based on the log-rank test). Our findings demonstrate that (Figure S2A) high CA7 score HNSCCs (n = 420) were associated with poorer survival (HR = 1.497; *p* = 0.0389) when compared with the low CA7 HNSCCs (n = 101). Interestingly, among OPSCC patients (n = 80; HPV-neg = 26 and HPV-pos = 54) we found that a high CA7 score was associated with poor overall survival (Figure S2B), regardless of HPV status (HR = 11.369; *p* < 0.0001).

CA7 score was not able to stratify HPV-neg HNSCCs into high- and low-risk subgroups significantly. Since HPV-pos and HPV-neg tumors are distinct disease entities, we designed a subtype-specific weighted gene expression signature based on the appropriately weighted expression of the CA7 genes in each subgroup. To develop this signature, the expression for each CA7 gene was split into high- versus low-expression subgroups through the optimization of the log-rank test statistic, and then the hazard ratio parameter estimate for the high expression group was determined. High-expression gene groups that had a negative impact on survival had a positive parameter estimate, while genes that correlated positively with good prognosis had a negative parameter estimate. The total weighted sum for each patient was generated by adding the parameter estimates for each gene which had above threshold expression (if they were in the low expression group, they were given a 0 for that gene weight). The cutoff between high- and low-weighted CA7 scores was optimized using log-rank statistics. Notably, this new model was able to stratify HPV-neg HNSCCs into high- and low-risk groups with higher significance (HR = 1.867; *p* < 0.001) (Figure S2C,D). Among HPV-neg OPSCCs, the high CA7 group (n = 6) showed a strong trend towards poorer overall survival (HR = 2.242; *p* = 0.113) when compared to the low CA7 group (n = 20) (data not shown), but owing to the small sample size, this difference did not attain achieve statistical significance.

### 2.3. Hypoxia Enhances CA in HPV-Neg OPSCCs via HIF-1α

**Figure 3:**
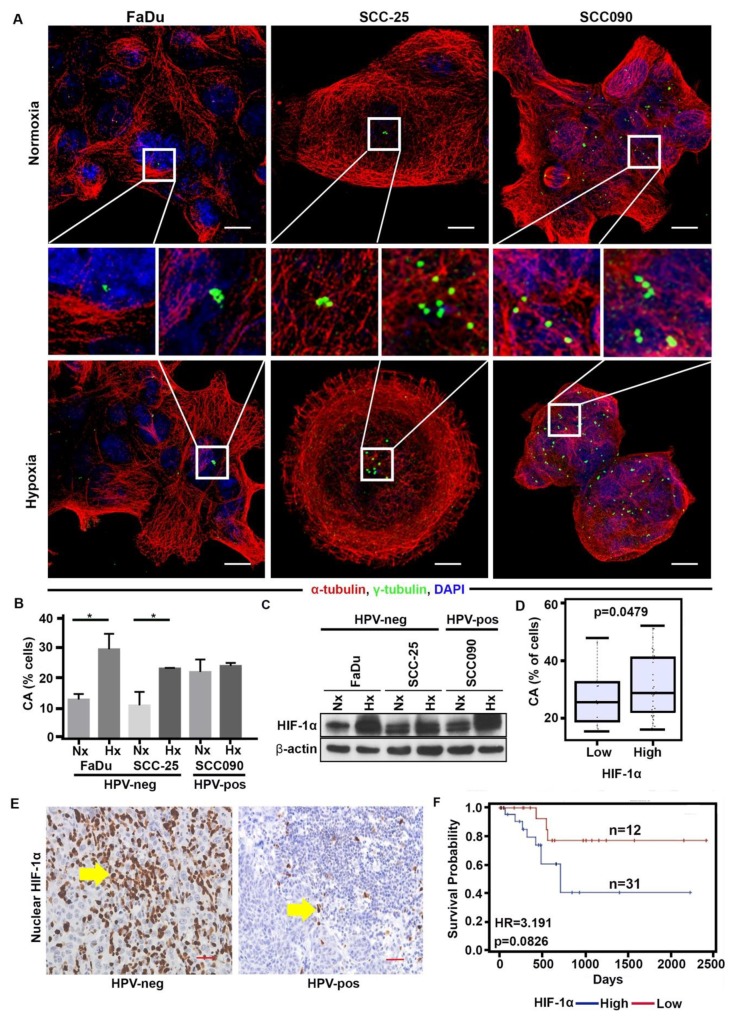
HPV-neg OPSCCs show a strong association between CA and HIF-1α expression. (**A**) Confocal immunomicrographs showing numerical CA in HPV-pos and HPV-neg tumor cells cultured in normoxic and hypoxic conditions. OPSCC cells were immunostained for centrosomes (γ-tubulin, green), microtubules (α- tubulin, red) and counterstained with Hoechst (blue). Scale bar (white), 20 μm. (**B**) Percentage distribution of cells with CA (structural and numerical) in HPV-neg and HPV-pos cells in normoxic (Nx) and hypoxic (Hx) conditions. (**C**) Immunoblots of HIF-1α in FaDu, SCC-25, and SCC090 OPSCC cells cultured in normoxic (Nx) and hypoxic (Hx) conditions. (**D**) Box plot depicting the distribution of CA in HIF-1α-high (n = 30) and -low (n = 12) HPV-neg tumors (*p* = 0.0479). (**E**) Representative immunohistochemical micrographs of HPV-pos and HPV-neg OPSCC tumors stained for HIF-1α. (**F**) Kaplan Meier survival analysis representing overall survival in HPV-neg OPSCCs stratified based on HIF-1α scores (HR = 3.191; *p* = 0.0826). * *p* < 0.05.

Our laboratory has previously identified that hypoxia induces CA in breast tumors via HIF-1α. Given that the discordance in CA is much higher between patient samples and cultured cell lines in HPV-neg OPSCCs compared to HPV-pos ones, we hypothesized that this discrepancy may be a result of the divergent tumor cell micro-environments under in vivo and in vitro conditions. Hypoxia, which is inadequately reflected in in vitro cell cultures, may underlie this observed divergence in CA. To test this hypothesis, we exposed HPV-neg (SCC-25 and FaDu) and HPV-pos (SCC90) OPSCC cell lines to hypoxia for 48 h using a hypoxic chamber flushed with a 1% O_2_ gas mixture. The presence of hypoxia was confirmed by the upregulation of HIF-1α through Western blot (Figure 3C and Figure S8 and Table S3). We observed that following hypoxia treatment, HPV-neg OPSCC cells (SCC-25 and FaDu) exhibited significantly higher CA (*p* < 0.05) compared to their normoxic controls, whereas no significant difference in CA levels was observed in HPV-pos OPSCC cells (SCC90) (Figure 3A,B). It is well-recognized that hypoxia mediates its function through the regulation of hypoxia-regulated genes by the transcription factor hypoxia-inducible factor-1 (HIF-1). The functional HIF-1 heterodimer (composed of alpha and beta subunits) is stabilized under hypoxic conditions and binds to hypoxia response elements (HREs) in target gene promoters. To confirm that the increase in CA under hypoxia was due to HIF-1α, cells cultured under normoxic conditions were transfected with GFP-tagged degradation-resistant HIF-1α (Figures S3C and S9 and Table S3). These transfected SCC25 and FaDu (HPV-neg) cell lines displayed higher CA (FaDu HIF-1α OE ~24%, SCC-25 HIF-1α OE ~22%) under normoxic conditions than vector controls (FaDu HIF-1α CV 12%, SCC-25 HIF-1α CV 9%) (Figure S3A,B) and the increase in CA was further confirmed with RT- PCR (Figure S3D). Meanwhile, no significant difference was observed between the control (control vector- CV) and HIF-1α overexpressing (overexpression- OE) HPV-pos OPSCC cells (SCC90).

Next, to corroborate our findings in the clinical samples, we asked whether the high-CA HPV-neg OPSCC patient samples also showed higher HIF-1α expression compared to the low-CA ones. For this, serial sections of the 87 OPSCC samples (used to quantify CA in Figure 1—due to the loss of tissue, eleven cases were not included in the HIF-1α analysis) were immunohistochemically stained for HIF-1α. The nuclear HIF-1α weighted index (WI) was calculated as indicated in Materials and Methods. The patients were stratified into high- and low- HIF-1α expressing subgroups using the optimal HIF-1α expression cut-point (based on the log-rank test). In the whole cohort, the expression of HIF-1α was positively correlated with the CA (R = 0.319; *p* = 0.03). Figure S4 shows that OPSCCs with high HIF-1α showed higher CA compared with low HIF-1α OPSCCs. Figure 3D depicts that HPV-neg OPSCCs with high HIF-1α (n = 30) showed higher CA (*p* = 0.0479) compared with low HIF-1α HPV-neg OPSCCs (n = 12). The survival analysis (Figure 3E,F) demonstrated that the high HIF-1α expressing HPV-neg OPSCCs were associated with poorer overall survival (HR = 3.191; *p* = 0.0826) than the low HIF-1α HPV-neg OPSCCs.

We further evaluated the differences in the expression of hypoxia-associated genes in HPV-neg and HPV-pos HNSCCs. A 26-gene hypoxic signature was probed in the same dataset (TCGA) used in Results Section 2 [26]. Interestingly, our results indicate significantly higher expression (*p* = 3.77 × 10^−7^) of the 26 hypoxia-associated genes *(ALDOA, ANGPTL4, ANLN, BNC1, C20ORF20, CA9, CDKN3, COL456, DCBLD1, ENO1, FAM83B, FOSL1, GNAL1, HIG2, KCTD11, KR717, LDHA, MPRS17, P4HA1, PGAM1, PGK1, SDC1, SLC16A1, SLAC2A1, TPI1, VEGFA)* and HIF-1α in HPV-neg (n = 422) head and neck tumors compared with those that were HPV-pos (n = 97) (Figure S5A). More so, in the analysis of the subset consisting of only OPSCCs among the cohort, we found similar results, wherein HPV-neg OPSCCs (n = 26) showed significantly (*p* < 0.001) higher expression of the hypoxia gene signature compared with the HPV-pos OPSCCs (n = 54) (Figure S5B). In addition, the hypoxia score was able to stratify the OPSCCs into high and low-risk groups. The high-hypoxia group was associated with significantly poorer overall survival when compared with the low-hypoxia group (HR = 3.297; *p* = 0.0127). Notably, among the HPV-neg OPSCCS, high-hypoxia HPV-neg OPSCCs exhibited poorer overall survival (HR = 2.197; *p* = 0.205) relative to the low-hypoxia HPV-neg OPSCCs (Figure S6). We also observed a positive correlation between the CA7 and the hypoxia 26 gene scores in HPV-neg OPSCCs (R = 0.34760; *p* = 0.0819).

Collectively, the findings from our clinical data and TCGA analysis confirm that HIF-1α and CA are positively correlated in OPSCCs with higher significance in the HPV-neg tumors. These results suggest that the CA observed in HPV-neg OPSCCs may be hypoxia-induced and may underlie their poorer prognoses relative to the HPV-pos ones.

### 2.4. HIF-1α Upregulation Is Associated with Downregulation of miR-34a, Increase in CA and Expression of Cyclin D1

Having established the relationship between hypoxia and CA in HPV-neg OPSCCs, we sought to delineate the possible mechanism through which HIF-1α induces CA in OPSCC. Studies have also shown that hypoxia and HIF-1α regulate a panel of miRNAs [27]. miRNAs regulate the expression of genes involved in many vital events related to angiogenesis, tumorigenesis and even CA in multiple malignancies, including head and neck cancer [28]. Through our in silico analysis of the publicly-available TCGA miRNA-seq data, we analyzed the differential expression of the top 19 CA-associated miRNAs (list and expression reported in Table S2) in HPV-neg vs. HPV-pos OPSCCs. Among these 19, 12 miRNAs were upregulated in HPV-pos and seven were upregulated in the HPV-neg OPSCCs. The significant overexpression of miR-34a in HPV-pos tumors compared to HPV-neg ones (*p* = 0.000248) was particularly interesting. CCND1 mRNA is a known target of miR-34a, which has been shown to downregulate cyclin D1 expression [29]. In line with this, we observed *CCND1* gene expression levels to be significantly downregulated in HPV-pos tumors (*p* = 9.88 × 10^−9^), with a negative correlation between miR-34a and *CCND1* (Figure S7) expression levels. Furthermore, it has been reported that HIF-1α represses the expression of miR-34a in p53 deficient cancer cells [30]. Intriguingly, we observed a significantly higher expression of HIF-1α in HPV-neg HNSCCs in the TCGA dataset.

**Figure 4:**
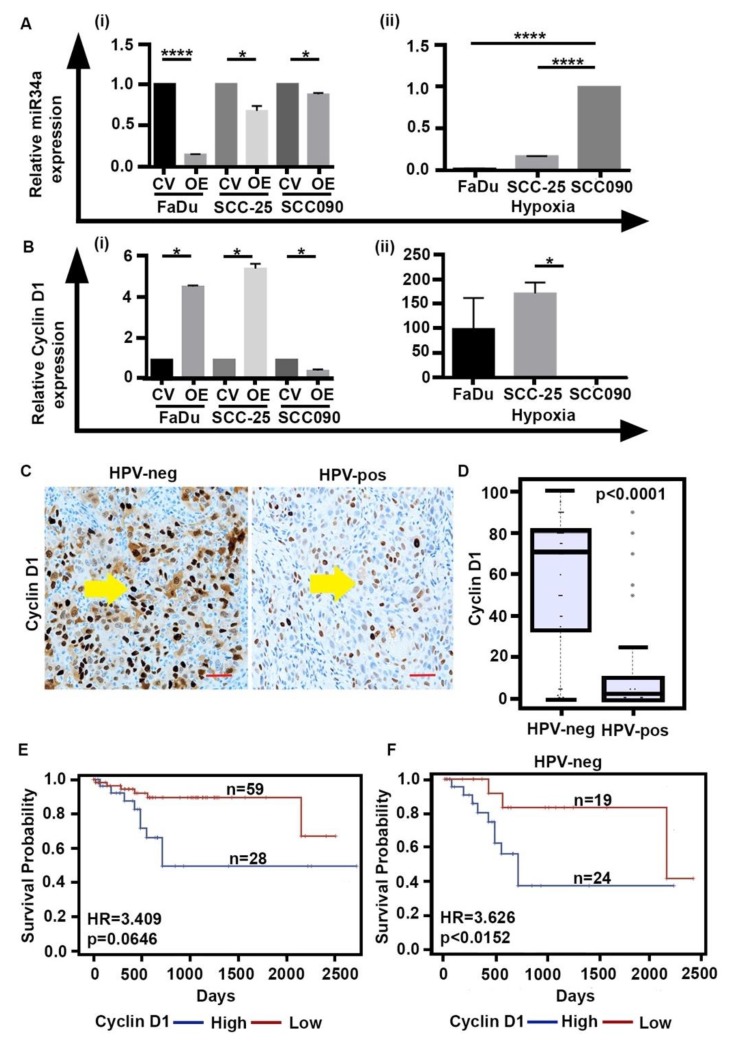
Hypoxia induces CA by upregulating the expression of cyclin D1 via downregulating miR-34a in HPV-neg OPSCCs. qRT-PCR analysis of (**Ai**) miR-34a in FaDu, SCC25, and SCC090 OPSCC cells cultured in normoxic and hypoxic conditions (normalized to normoxic conditions for each individual cell line), (**Aii**) miR-34a in FaDu, SCC25, and SCC090 OPSCC cells grown under hypoxic conditions compared to the expression of miR-34a in FaDu, SCC25, and SCC090 OPSCC cells grown under normoxic conditions, (**Bi**) Cyclin D1 in FaDu, SCC25, and SCC090 OPSCC cells cultured in normoxic and hypoxic conditions (normalized to normoxic conditions for each individual cell line), (**Bii**) Cyclin D1 in FaDu, SCC25, and SCC090 OPSCC cells cultured in hypoxic conditions. Data were normalized by the amount of Ctrl_miRTC_1 and beta-actin mRNA, for miR34a and *CCND1*, respectively, expressed relative to the corresponding value for all the cells and are means ± SD from triplicate data. (**C**) Immunohistochemical micrographs of HPV-pos and HPV-neg OPSCC tumors labeled with nuclear cyclin D1. Scale bar (red), 50 μm (**D**) Box plot representing the distribution of cyclin D1 WI in HPV-pos (n = 44) and HPV-neg (n = 43) tumors (*p* < 0.0001). (**E**) Kaplan Meier survival curves representing the overall survival of cyclin D1 high (n = 28) and low (n = 59) groups in HPV-neg and HPV-pos OPSCC patients (HR = 3.409; *p* = 0.0646). (**F**) Kaplan Meier survival curve representing the overall survival of cyclin D1-high (n = 24) and -low (n = 19) groups in HPV-neg OPSCC patients (HR = 3.626; *p* = 0.0152). * *p* ≤ 0.05, **** *p* ≤ 0.0001.

Based on these in silico findings, we hypothesized that hypoxia may induce the expression of CA-associated genes through the regulation of miR-34a in HPV-neg OPSCCs. To test this hypothesis, we performed quantitative PCR to evaluate the levels of miRNA-34a in HPV-pos and HPV-neg cells, both in control vector- and HIF-1α-overexpressing cells. Interestingly, in line with published studies, we observed that in all the cells, regardless of HPV status, HIF-1α overexpression negatively correlated with the levels of miRNA-34a, and this effect was more pronounced in HPV-neg cells (Figure 4A(i,ii)) than in HPV-pos cells. This decrease in miRNA-34a inversely correlated with the expression of *CCND1* which encodes cyclin D1 (which otherwise was not significantly different in the OPSCC cells) (Figure 4Bi). More so, cyclin D1 expression only increased in HPV-neg OPSCC cells (Figure 4Bii). Collectively, we observed that in HPV-neg OPSCC tumors, CCND1 expression is associated with HIF-1α upregulation and the downregulation of miR-34a, and an increase in CA.

To further bolster our in silico and in vitro findings, we examined the relationship of HIF-1α and cyclin D1 in clinical samples. To this end, we immunohistochemically stained the serial sections of the 87 OPSCC samples used in Results Section 1 and Section 2. Nuclear cyclin D1 WI was calculated as indicated in Materials and Methods. We found that cyclin D1 expression was significantly (*p* = 0.0001) higher in the HPV-neg (n = 43) OPSCCs when compared with the HPV-pos (n = 44) OPSCCs (Figure 4A,B) and high cyclin D1 expression was associated with poorer overall survival in OPSCCs (HR = 3.409; *p* = 0.0646) (Figure 4C). These findings are in line with the previous studies that showed a low expression of Cyclin D1 in HPV-pos HNSCCs (p16 inhibits cyclinD1-CDK4/6 (cyclin-dependent kinase 4/6) complexes). Also, cyclin D1 further stratified with HPV-neg OPSCCs in high- and low- risk groups (HR = 3.62; *p* = 0.0152) (Figure 4D). We also observed a strong positive correlation between HIF-1α and cyclin D1 scores in HPV-neg OPSCCs (Spearman’s rho ρ = 0.642, *p* < 0.001). Several studies have confirmed the overexpression of cyclin D1 in promoting CA, aneuploidy, and tumorigenesis [31]. In line with this, we also observed that the percentage of CA in HPV-neg OPSCC samples was positively correlated with cyclin D1 expression (Spearman’s rho ρ = 0.637, *p* < 0.001. Thus, these findings substantiate the paradigm that hypoxia induces CA in HPV-neg OPSCCs, at least in part, by the overexpression of cyclin D1.

Finally, we wanted to gain insight into the most informative biomarker for clinical decision making. We thus asked which biomarker (CA, HIF-1α, or cyclin D1) was best able to stratify HPV-neg OPSCCs into high- and low-risk groups. First, we performed a multivariate analysis, and noted that only CA showed a significant association with poor overall survival when other confounding factors like stage, therapy, gender, alcohol consumption, as well as expression levels of HIF-1α and cyclin D1, were taken in account (HR = 4.43; *p* = 0.062) (Table 3A). Next, to measure the performance of the prognostic models, we used a measure of model fit, 2 Log Likelihood (−2LogL) (the model that minimized the −2LogL was considered superior). The results from this statistical test indicate that CA is the best-fit model (Table 3B). Similarly, when we performed the same test for our in silico findings, the weighted CA7 score better stratified the HPV-neg HNSCCs in high- and low-risk groups than the hypoxia score (Table 3C). Thus, collectively, these findings suggest that CA can serve as a clinically-informative phenotypic biomarker for the identification of high-risk HPV-neg OPSCC patients and can potentially also serve as a novel therapeutic target for these patients.

## 3. Discussion

CA, a key driver of CIN and an early driver of intratumoral heterogeneity [32], is associated with tumorigenesis and tumor progression in multiple cancers, including that of the head and neck. Previous studies in surgically resected HNSCCs have shown an association between higher CA and local recurrence, with CA being a better predictor of recurrence than other commonly used parameters such as T stage [33,34]; furthermore, higher CA has been linked with poor overall survival in HNSCCs [35]. Despite this, no rigorous comparison of CA in OPSCCs that differ in their HPV status has been performed to date. Neither has the prognostic potential of CA in OPSCCs been investigated. The generation of CA in HPV-pos HNSCCs is known in greater detail and is related to HPV infection, jump-started by the viral oncoproteins (E6 and E7). For a long time, the comparatively lower CA observed in in vitro HPV-neg HNSCC cell lines was presumed to be due to the absence of the drivers E6 and E7. In fact, the HPV-neg HNSCCs display a CA driving mechanism distinct from that of HPV-pos HNSCCs, which lacks a detailed description. To shed light on this subject, we performed rigorous quantitation of CA (structural and numerical centrosomal aberrations) in a large cohort of HPV-neg and HPV-pos OPSCC tumor samples and explored the role of hypoxia, a hitherto overlooked driver of CA, in the generation of CA in HPV-neg tumors.

The findings from this study uncover that HPV-neg OPSCCs exhibit higher CA than HPV-pos OPSCCs, and that CA was associated with poorer overall survival in HPV-neg OPSCCs, even when all the other confounding factors were controlled for. In addition, higher CA was associated with poorer overall survival in OPSCCs, regardless of HPV status. These findings were corroborated by our in silico analysis of CA-associated genes in the large, well-annotated TCGA microarray dataset (Figure 2). Since CA can be induced by perturbations in the expression of many different genes, we focused on a set of seven genes commonly associated with centrosome structure/biogenesis and whose dysregulation is known to induce CA. Impressively, this “CA7” gene signature was significantly prognostic in HNSCCs as well as in OPSCCs, and was able to stratify HPV-neg HNSCCs into high and low-risk groups.

Multiple factors are responsible for differences in the biology of HPV-neg and HPV-pos OPSCCs, the prime among which is a varied tumor microenvironment [36]. Hypoxia is a classic feature of the tumor microenvironment that makes tumors more resistant to treatments and is associated with poor prognosis across a variety of cancers. In accordance with this, we observed higher HIF-1α expression in the HPV-neg OPSCCs [37]. Also, high HIF-1α expression strongly correlated with high CA in HPV-neg OPSCCs. Previously, however, separate studies have reported conflicting results regarding HIF-1α expression in HPV-pos versus HPV-neg tumors [38,39]. Other endogenous hypoxia markers such as CA IX have also not yielded definitive results [40]. Scrutinizing a panel of hypoxia markers rather than relying on individual ones can provide a far more comprehensive picture of the hypoxic environment in HNSCCs as well as the intratumoral heterogeneity and cellular responses it drives. Therefore, in this study, a previously established 26-gene hypoxia signature with addition of HIF-1α gene expression was used in in silico analyses of the publicly-available TCGA dataset, revealing higher expression of hypoxia-associated genes in HPV-neg OPSCCs, which correlated with poorer overall survival within this subset. The observation that hypoxia and the resulting HIF-1α activation can induce CA provides a compelling explanation for the higher CA observed in our HPV-neg OPSCC clinical samples, and consequently the poorer survival, compared with HPV-pos OPSCCs.

The analysis of miRNA expression in OPSCC has revealed a possible mechanistic link between hypoxia and CA. In this study, we have newly identified miR-34a as a probable player in driving CA in OPSCC. The dysregulation of miR-34a has been observed in different types of cancers. For example, by targeting *CCND1*, miR-34a controls the expression of cyclin D1, a protein whose upregulation triggers CA. Since hypoxia through HIF-1α has been shown to repress the expression of miR-34a [41], a downregulation of miR-34a in HPV-neg tumors leads to cyclin D1 overexpression and CA. Our in vitro findings are commensurate with this observation, wherein clinical samples of HPV-neg OPSCCs expressed higher levels of cyclin D1 and higher CA, and was associated with poor overall survival in these patients. Therefore, our findings indicate a HIF-1α-mediated downregulation of miR-34a in HPV-neg tumors which drives its distinct tumor biology and establishes a causative link between hypoxia and CA, which co-occur in many solid tumors.

Studies have shown that EGFR inhibitors such as cetuximab are effective in treating HPV-neg HNSCCs [42]. However, only a modest effect on survival has been shown when cetuximab was co-administered with conventional chemotherapy. Therefore, new molecular targets are required to improve survival in HPV-neg HNSCCs. Our study has uncovered CA as an objectively evaluable and actionable phenotypic biomarker and has yielded novel insights into potential therapeutic targets for HPV-neg OPSCCs.

## 4. Materials and Methods

### 4.1. Clinical Tissue Samples

Formalin-fixed paraffin-embedded OPSCC tissue microarrays (TMA) of tonsil, base of tongue, and soft palate tumors were procured from the Greater Poland Cancer Centre, Poznan in Poland. Patients were diagnosed between the years 2007 and 2014, and based on the location of tumor (tonsil, base of tongue and soft palate) the patients were selected for the TMA construction. Clinicopathologic characteristics of the patients are provided in Table 1. The Institutional Review Board of Greater Poland Cancer Centre, Poznan, PL approved all aspects of the study (The IRB code: 412/18). The methods were carried out in accordance with the approved guidelines stipulated in the Material Transfer Agreements and Data User Agreements between Greater Poland Cancer Centre, Poznan, PL, and Georgia State University. Informed consent was obtained from all subjects.

### 4.2. HPV Analysis

The detection of high-risk HPV was performed using GP5+/GP6+ HPV DNA PCR with enzyme-immunoassay (EIA). For the genotyping of the viral DNA, the Luminex platform was used for bead-based array. The EIA detected 14 HPV types: 16, 18, 31, 33, 35, 39, 45, 51, 52, 56, 58, 59, 66, 68. β-globin PCR was used to test for sample quality post-DNA extraction [43].

### 4.3. Immunohistofluorescence Staining and Quantitation

The TMA slides were deparaffinized as described previously [21]. Antigen retrieval was performed by heating in a pressure cooker with citrate buffer (pH 6.0). The tissues were blocked with 5% BSA in PBS solution for 30 min. After blocking, primary antibody incubation with γ-tubulin (Sigma, St. Louis, MO, USA) at a dilution of 1:1000 was performed for 1 h at room temperature. The tissues were then washed 3× with PBS after which incubation with secondary antibody (Alexa-488 anti-mouse) was performed at room temperature for 1 h at a dilution of 1:2000. After 3× washes in PBS, coverslips were mounted with Prolong-Gold Antifade with DAPI (Invitrogen, Waltham, MA, USA). Tissue sections were imaged using the Zeiss LSM 700 confocal microscope (Ziess, Oberkochen, Germany). Details on imaging and analysis are included in Supplementary Materials.

### 4.4. Immunohistochemistry of Cyclin D1 and HIF-1α and Scoring

The initial steps from deparaffinization to antigen retrieval were performed as described for immunofluorescence. The tissues were then blocked with Ultravision Protein Block (ThermoFisher, Waltham, MA, USA) for 30 min followed by hydrogen peroxide block with Ultravision Hydrogen Peroxide Block (ThermoFisher) for 10 min. The tissues were then incubated with antibodies directed against HIF-1α (Abcam, Cambridge, MA, USA) or cyclin D1 (ThermoFisher) at a dilution of 1:1000 for 1 h. After 3× washes in TBST, the slides were subjected to secondary antibody incubation using anti-Rabbit HRP (Biocare, Pacheco, CA, USA) for 1 h. Enzymatic detection was performed using DAB Chromogen Kit (Biocare). Nuclear HIF-1α and cyclin D1 staining were categorized as 0 = none, 1 = low, 2 =moderate, and 3 = high. The percentage of positive cells, defined as cells with a staining intensity of 1+, quantitated from a total of around 500 cells, was determined. The weighted index (WI) for each sample was calculated as the product of the percentage of cell positivity and staining intensity.

### 4.5. Cell Culture and Hypoxia Treatment

FaDu, SCC25 (HPV-negative) and SCC90 (HPV-positive) cells were purchased from American Type Culture Collection, Manassas, VA. FaDu cells were maintained in EMEM medium supplemented with 10% FBS and 1% penicillin/streptomycin. SCC25 cells were maintained in 1:1 mixture of DMEM and Ham’s F12 medium supplemented with 400 ng/mL hydrocortisone, 10% FBS and 1% penicillin/streptomycin. SCC 90 cells were maintained in EMEM supplemented with 2mM L-glutamine, 10% FBS, and 1% penicillin/streptomycin. All the cell lines were cultured under standard culture conditions—37 °C and 5% CO_2_. For hypoxia induction, the cells were placed in a hypoxic modulated incubator chamber which was flushed with 5% CO_2_ and a gas mixture containing 1% CO_2_ and 94% N_2_ gas mixture at 20 L/min for 7–10 min every 3–6 h. HIF-1α was genetically overexpressed by transfecting cells with GFP-tagged degradation resistant HIF-1α. HA-HIF-1α P402A/P564A-pcDNA3 was a generous gift from Dr. Willian Kaelin (Addgene plasmid # 18955). Cells at a confluency of ~70% were transfected using Lipofectamine LTX according to the manufacturer’s instructions.

### 4.6. RNA Extraction and Quantitative Real Time PCR

The total RNA from the cell lines after transfection and hypoxia induction was extracted using TRIzol reagent (Takara Bio, Inc., Otsu, Japan). Genomic DNA contamination was eliminated from RNA preparation by digesting with RNase-free DNase (Qiagen). cDNA was prepared using iScript™ cDNA Synthesis Kit (Bio-Rad). All PCR reactions were performed using the fluorescent SYBR Green methodology. Quantitative RT-PCR (qRT-PCR) was run in Quant Studio 3 Real-Time PCR system (Thermo Fisher Scientific, Inc.) with SYBR-Green PCR Master Mix (ThermoFisher Scientific) following the manufacturer’s protocol. The relative quantification was calculated by the 2^−ΔΔCt^ method. The expression of all mRNAs was normalized with respect to GAPDH or beta-actin.

### 4.7. miRNA Extraction and Quantitative Real Time PCR

The total RNA was isolated from the cell lines after transfection and hypoxia induction using miRNeasy Micro Kit (Qiagen) per the manufacturer’s protocol. Genomic DNA contamination was eliminated by digesting the RNA with RNase-free DNase (Qiagen). cDNA was prepared using the miScript II RT Kit (Qiagen). qRT-PCR was performed using the miScript SYBR green PCR kit, and specific miRNA primer assays (Qiagen) following the manufacturer’s protocol. qRT-PCR was run in Quant Studio 3 Real-Time PCR system (Thermo Fisher Scientific, Inc.) and relative expression was quantitated using the 2^−ΔΔCt^ method. The expression of mir-34 A was normalized to Ctrl_miRTC_1.

### 4.8. Immunocytofluorescence Staining

HPV-pos and HPV-neg cells were grown on poly-l-lysine-coated cover glasses. Following hypoxia treatment, the cells were fixed with ice cold methanol for 10 min. After sequentially blocking in PBS with 5% BSA and 10% goat serum for 30 min, the cells were incubated with primary antibodies directed against α-tubulin and γ-tubulin (1:1000 dilution) prepared in 1% BSA-PBS (with 0.1% Triton X-100) at 37 °C for 35 min. Following primary antibody incubation, the cells were washed five times with PBS before adding the secondary antibody. The cells were incubated with Alexa 555-and 488-conjugated antibodies (Invitrogen) at 37 °C for 35 min, after which the cells were washed with PBS for 10 min. For DNA visualization, the cells were stained with Heochst33342 (Invitrogen) and then mounted onto glass slides with ProLong Gold Antifade Reagent. Fluorescent images were captured using the Zeiss LSM 700 confocal microscope (Oberkochen, Germany) and processed with Zen software (Oberkochen, Germany).

### 4.9. Statistical Analyses

For clinical data, as well as our in silico data analysis, patients’ overall survival was used as the endpoint for survival analysis. A log-rank test was applied to determine the significance of survival differences between subgroups. The cut-off points that we found for CA and HIF1-α were those which maximized survival differences between high- and low-risk subgroups. The range of CA value was 4.85–71.31 and 23 was used as the cut-off point as it resulted in the minimization of the log-rank p-value. The test of group mean differences shown in box-whisker plots is based on the Mann–Whitney U test. In cases with more than two groups, the differences were evaluated by the Kruskal–Wallis test. Statistical analyses were performed using SAS software 9.4 (SAS Institute Inc., Cary, NC). Details of the survival model used in our in silico analysis are included in the Supplementary Materials.

## 5. Conclusions

This is the first report to substantiate the previously unrecognized role of HIF-1α-induced CA in HPV-neg OPSCCs, revealing a molecular pathway that may be responsible for the CIN, intratumoral heterogeneity, and poor prognosis associated with these tumors. The prognostic potential of CA is especially resounding within HPV-neg OPSCCs, facilitating the enhanced identification of higher risk patients, influencing future treatment strategies, and providing a platform for the discovery of effective molecular targets.

## Figures and Tables

**Table 1:** Descriptive statistics of clinicopathological characteristics and treatment for OPSCC patients in the clinical samples analyzed for centrosome amplification phenotypes.

Baseline Characteristics	HPV-Neg	HPV-Pos	*p* Value
**Gender, n (%)**			
Female	11 (21.57)	19 (40.43)	0.0430
Male	40 (78.43)	28 (59.57)	
**Tumor Grade, n (%)**			
1	5 (9.80)	1 (2.13)	0.2957
2	22 (43.14)	27 (57.45)	
3	18 (32.29)	13 (27.66)	
N/A	6 (11.76)	6 (12.77)	
**Tumor Stage, n (%)**			
II	1 (1.96)	0 (0.00)	0.1933
III	15 (29.41)	9 (19.15)	
IV	28 (54.90)	35 (74.47)	
N/A	7 (13.73)	3 (6.38)	
**Smoking, n (%)**			
0	6 (11.76)	5 (10.64)	<0.0001
1	3 (5.58)	29 (61.70)	
2	4 (7.84)	6 (12.77)	
3	38 (74.51)	7 (14.89)	
**Alcohol, n (%)**			
No	42 (82.35)	46 (97.87)	0.0393
Yes	8 (15.69)	1 (2.13)	
N/A	1 (1.96)	0 (0.00)	
**CA, n (%)**			
High	34 (66.67)	26 (55.32)	0.2494
Low	17 (33.33)	21 (44.58)	
**Chemotherapy, n (%)**			
None	32 (62.75)	18 (38.30)	0.0070
Concomitant	12 (23.53)	26 (55.32)	
Neoadjuvant	2 (3.92)	0 (0.00)	
Adjunctive	2 (3.92)	0 (0.00)	
N/A	3 (5.88)	3 (6.39)	
**Radiotherapy, n (%)**			
None	5 (9.80)	2 (4.26)	0.1828
Primary	11 (21.57)	14 (29.79)	
Adjuvant	26 (50.98)	29 (61.70)	
Palliative	6 (11.76)	1 (2.13)	
N/A	3 (5.88)	1 (2.13)	

**Table 2:** Univariate and multivariate Cox proportional regression analysis for overall survival in HPV-neg OPSCCs, comparing the influence of common clinicopathological variables relative to the percentage of CA on patients’ overall survival.

Variable		Univariate Analysis				Multivariate Analysis			
		*p*-Value	Hazard Ratio	95% Hazard Ratio Confidence Limits		*p*-Value	Hazard Ratio	95% Hazard Ratio Confidence Limits	
**CA**	High vs. low	0.05	5.38	1.69	42.17	0.02	7.38	1.32	41.28
**Gender**	Female	0.35	0.37	0.05	2.90	0.61	1.64	0.23	11.49
**Age at diagnosis**	High vs. low	0.37	0.96	0.86	1.06	0.27	1.09	0.93	1.29
**Overall stage**	IV vs. other	0.77	0.84	0.27	2.64	0.49	1.87	0.32	11.04
**Alcohol abuse**	Yes vs. no	0.46	1.79	0.38	8.37	0.18	7.12	0.39	129.29
**Radiotherapy**	Yes	0.81	1.29	0.16	10.20	0.99	0.00	0.00	-
**Smoking**	No vs. yes	0.95	0.96	0.26	3.57	0.85	1.16	0.24	5.69
**Tumor size**	>vs. ≤4	0.35	1.86	0.50	6.88	0.56	0.58	0.09	3.62
**Nodal metastasis**	Present vs. absent	0.94	0.92	0.11	7.85	0.17	0.29	0.05	1.71

**Table 3:** Multivariate analysis for HPV-neg OPSCCs comparing the high- and low-CA group. (**A**) −2log L model fit test for clinical samples. (**B**) and (**C**) −2log L model fit test for in silico TCGA dataset.

**A**			
**Variables**		**Multivariate Analysis**	
		***p*-Value**	**Hazard Ratio**
**CA**	High vs. low	0.063	4.433
**Gender**	Male	0.939	1.070
**Age at diagnosis**	High vs. low	0.227	1.076
**Overall stage**	IV	0.852	1.163
**Alcohol abuse**	Yes vs. no	0.166	0.160
**Radiotherapy**	Yes	0.994	0.000
**HIF-1α**	Yes	0.994	0.991
**Cyclin D1**	Yes	0.287	3.343
**B**			
**Variable**	**Cyclin D1**	**HIF-1α**	**CA**
**−2 Log L**	74.741	76.340	74.547
**C**			
**Variable**	**−2 Log L**		
**Null**	1912.9		
**Weighted Index of CA7**	1897.6		
**Basic Sum CA7**	1911.0		
**Hyp26 Score**	1911.9		
**CCNDI**	1907.1		

## Supplementary Materials

The following are available online at https://www.mdpi.com/2072-6694/12/2/517/s1, Figure S1: Analysis of CA in OPSCC clinical samples. Figure S2: Upregulation of CA7 genes is associated with poor overall survival in HNSCCs. Figure S3: (A) Confocal immunomicrographs showing numerical CA in HPV-pos and HPV-neg OPSCC cells transfected with empty vector or degradation-resistant HIF-1α. OPSCC cells were immunostained for centrosomes (γ-tubulin, green), microtubules (α- tubulin, red) and counterstained with Hoechst (blue). Scale bar (white), 20 μm. (B) Quantitation of centrosome aberrations per microscopic examination for HPV-pos and HPV-neg OPSCC cells transfected with empty vector or degradation-resistant HIF-1α. (C) Immunoblots of HIF-1α in FaDu, SCC-25, and SCC090 OPSCC cells transfected with degradation-resistant HIF-1α and control vector. (D) qRT- PCR analysis of mRNAs for γ-tubulin, pericentrin, centrin-1, and PLK4 in FaDu, SCC-25, and SCC090 OPSCC cells transfected with empty vector or degradation-resistant HIF-1α. Data were normalized by the amount of beta-actin mRNA, expressed relative to the corresponding value for all the cells and are means ± SD from triplicate data. Figure S4: Box plot depicting the distribution of CA in HIF-1α-high (n = 40) and -low (n = 47) OPSCCs (*p* = 0.0007). Figure S5: Comparison of 26-gene hypoxia gene signature in HPV-pos versus HPV-neg HNSCC tumors. Figure S6: (A) and (B) Kaplan Meier survival curves representing the survival probabilities of high and low hypoxia score OPSCC patients (HR = 3.297; *p* = 0.0127) and the survival probabilities of high and low hypoxia score hypoxia HPV-neg OPSCC patients (HR = 2.197; *p* = 0.2050), respectively. Figure S7: *CCND1* gene expression is negatively correlated with the miR-34a expression in HPV-pos HNSCCs. Figure S8: Whole blot showing all the bands with all molecular weight markers are shown for Figure 3C. Figure S9: Whole blot showing all the bands with all molecular weight markers are shown for Figure S3C. Table S1: Descriptive statistics of clinicopathological characteristics for HNSCC patients in the cohort (TCGA) used for in silico analysis of the prognostic value of CA7 signature. Table S2: List of CA associated miRNAs sorted by logFC values. Table S3: Densitometry values relative to loading control β-actin calculated using Image-J for immunoblot assays provided in main manuscript and supplementary data.
