# Supplementary material for: Hypoxia-Induced Centrosome Amplification Underlies Aggressive Disease Course in HPV-Negative Oropharyngeal Squamous Cell Carcinomas
Source: Cancers (Basel). 2020 Feb 24;12(2):517. doi: 10.3390/cancers12020517 (PMC7072660; doi:10.3390/cancers12020517)
Supplement: Supplementary file 1 [file cancers-12-00517-s001.pdf]

Hypoxia-Induced Centrosome Amplification Underlies Aggressive Disease Course in HPV-Negative Oropharyngeal Squamous Cell Carcinomas

Karuna Mittal, Da Hoon Choi, Guanhao Wei, Jaspreet Kaur, Sergey Klimov, Komal Arora, Christopher C. Griffith, Mukesh Kumar, Precious Imhansi-Jacob, Brian D. Melton, Sonal Bhimji-Pattni, Remus M. Osan, Padmashree Rida, Paweł Golusinski and Ritu Aneja

Supplementary Materials and Methods

Quantification of both structural and numerical centrosome amplification

We define CA as both structural and numerical aberration of centrosomes. TMAs immunostained for γ-tubulin (centrosomes, red) and DAPI (nuclei, blue) were observed with Zeiss LSM 700 confocal microscope (Ziess, Oberkochen, Germany) for centrosome amplification. At least 10 fields were imaged at 63X to capture enough nuclei and centrosomes. Within each field, we selected regions containing distinguishable nuclei and quantified the number and volume of γ-tubulin foci for each nucleus to assess numerical and structural CA, respectively. 250 nuclei in total were counted at random using Imaris software that allowed us to visualize the nuclei and centrosomes in 3D. We defined "numerical" amplification as interphase nuclei associated with more than two centrosomes, and "structural" amplification was defined as nuclei associated with at least one centrosome that had a volume greater than 0.7 μm^3. This threshold value was determined after past studies in our lab that defined the normal volumetric range of centrosomes across different cancer types, including breast, pancreas, bladder and OPSCCs, in which 500 centrosomes for each cancer type were counted from tissues of adjacent uninvolved cancer patients and normal tissues. Then an average normal range of volumes was determined, and centrosomes with a volume greater than this range were considered to be abnormal. Then, we independently counted cells (nuclei) that harbored numerical amplification of centrosomes and cells that harbored structural centrosome amplification from our total of 250 cells/nuclei. Percentage of cells with CA for each of our OPSCC samples was obtained by adding percentage of cells containing either structurally amplified centrosomes or numerically amplified centrosomes, or both.

Survival model analysis

For the survival model used in *in silico* analysis with individual genes, each gene was split into a high and low expression group by optimizing the log-rank test statistic, and the hazard ratio parameter estimate for the high expression group was determined. High expression gene groups with negative impact on survival had a positive parameter estimate while genes whose expression correlated positively with survival had a negative parameter estimate. The total weighted sum, for each patient, was generated by adding the parameter estimates for each gene that had above-threshold expression (if they were in the low expression group they were given a 0 for that gene weight). The cutoff between high and low weighted scores was similarly derived by finding the optimal log-rank statistic.

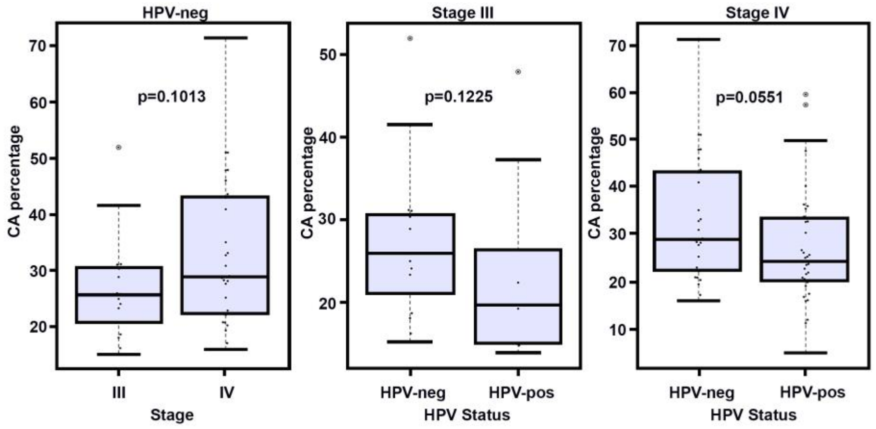

Supplementary Figure 1: Analysis of CA in OPSCC clinical samples. (A) Distribution of CA between stage III (n=15) and stage IV (n=28) in HPV-neg OPSCCs (p=0.1013). (B) Distribution of CA between HPV-neg (n=15) and HPV-pos (n=9) stage III patients (p=0.1225). (C) Distribution of CA between HPV-neg (n=28) and HPV-pos (n=35) stage IV patients (p=0.0551). Distributions for stage I (n=0) and II (n=1) were not depicted owing to lack of patients in that category.

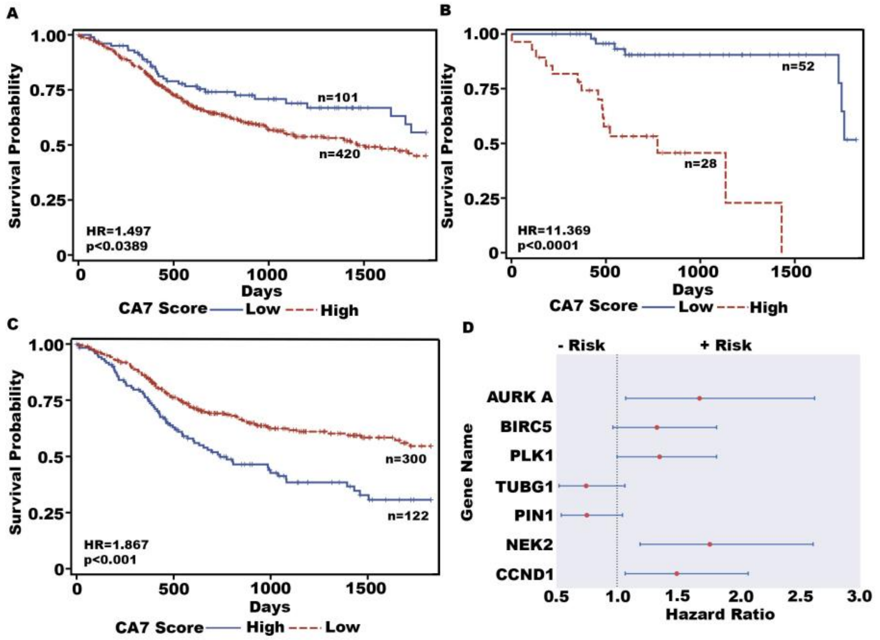

Supplementary Figure 2: **Upregulation of CA7 genes is associated with poor overall survival in HNSCCs**. **(A)** Kaplan Meier survival curves representing the survival probability of HNSCC patients stratified according to CA7-high (n=420) and CA7-low (n=101) expression levels (HR=1.497; p=0.0389). **(B)** Kaplan Meier survival curves representing the survival probability of OPSCC patients stratified according to CA7-high (n=28) and CA7-low (n=52) expression levels (HR=11.369; p<0.0001). **(C)** Kaplan Meier survival curve of HPV-neg HNSCC patients stratified according to the weighted CA7-high (n=122) and CA7-low (n=300) expression levels (HR=1.867; p<0.001). **(D)** Forest plot of hazard ratios for each of the CA7 genes in HPV-neg HNSCCs. The blue lines represent 95% confidence intervals. The HR for each gene is considered to be significant (p<0.05) if the blue lines do not cross HR of 1.0.

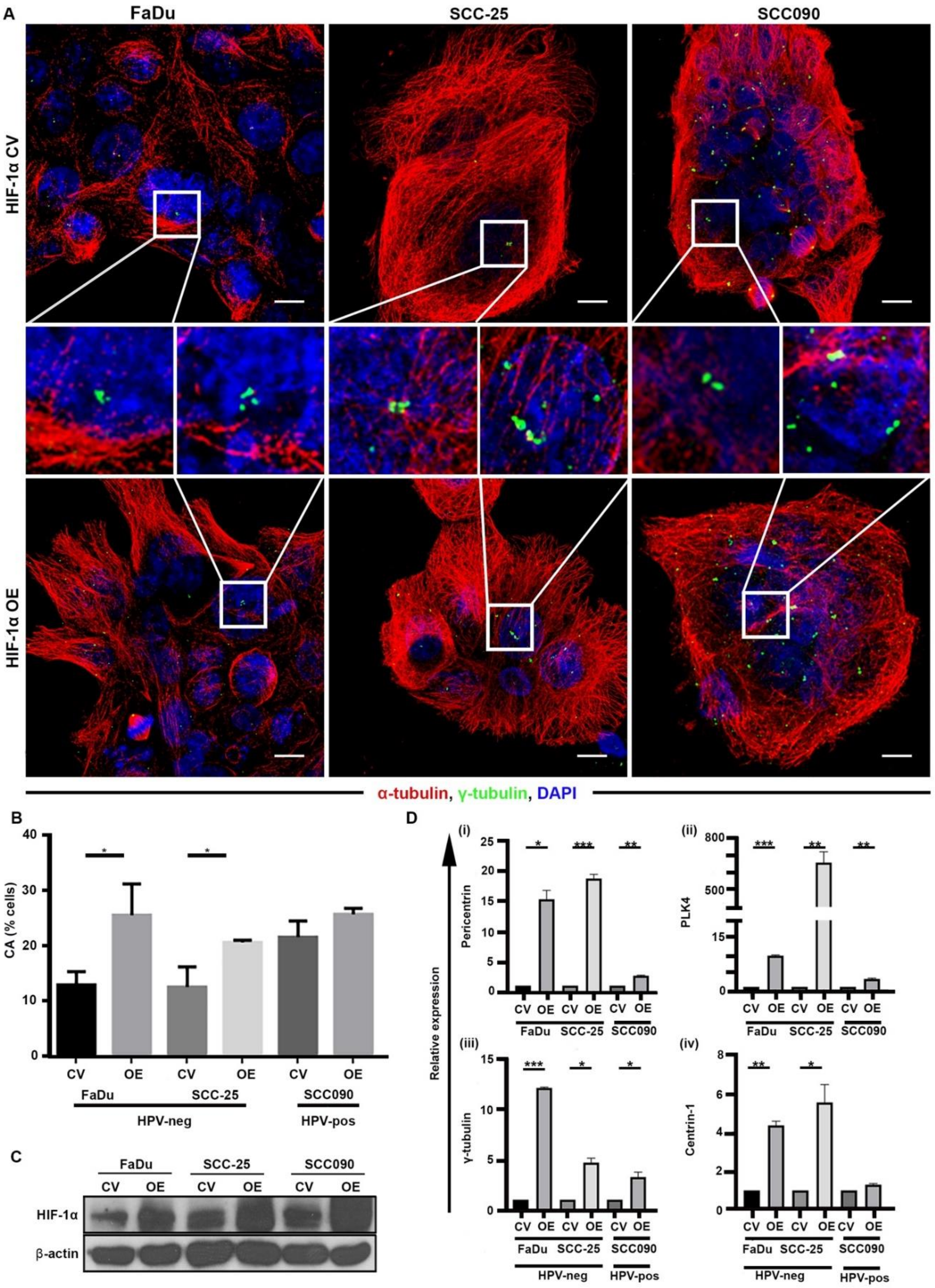

Supplementary Figure 3: (A) Confocal immunomicrographs showing numerical CA in HPV-pos and HPV-neg OPSCC cells transfected with empty vector or degradation-resistant HIF-1α. OPSCC cells were immunostained for centrosomes (γ-tubulin, green), microtubules (α -tubulin, red) and counterstained with Hoechst (blue). Scale bar (white), 20μm. **(B)** Quantitation of centrosome aberrations per microscopic

examination for HPV-pos and HPV-neg OPSCC cells transfected with empty vector or degradation-resistant HIF-1α. **(C)** Immunoblots of HIF-1α in FaDu, SSC-25, and SSC090 OPSCC cells transfected with degradation-resistant HIF-1α and control vector. **(D)** qRT- PCR analysis of mRNAs for γ-tubulin, pericentrin, centrin-1, and PLK4 in FaDu, SSC-25, and SSC090 OPSCC cells transfected with empty vector or degradation-resistant HIF-1α. Data were normalized by the amount of beta-actin mRNA, expressed relative to the corresponding value for all the cells and are means ± SD from triplicate data.

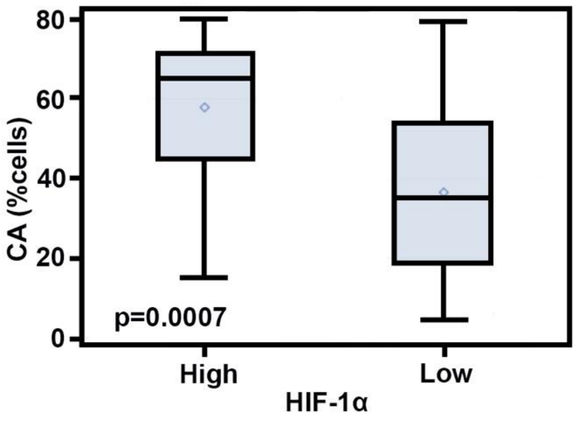

Supplementary Figure 4: Box plot depicting the distribution of CA in HIF-1α-high (n=40) and -low (n=47) OPSCCs (p=0.0007).

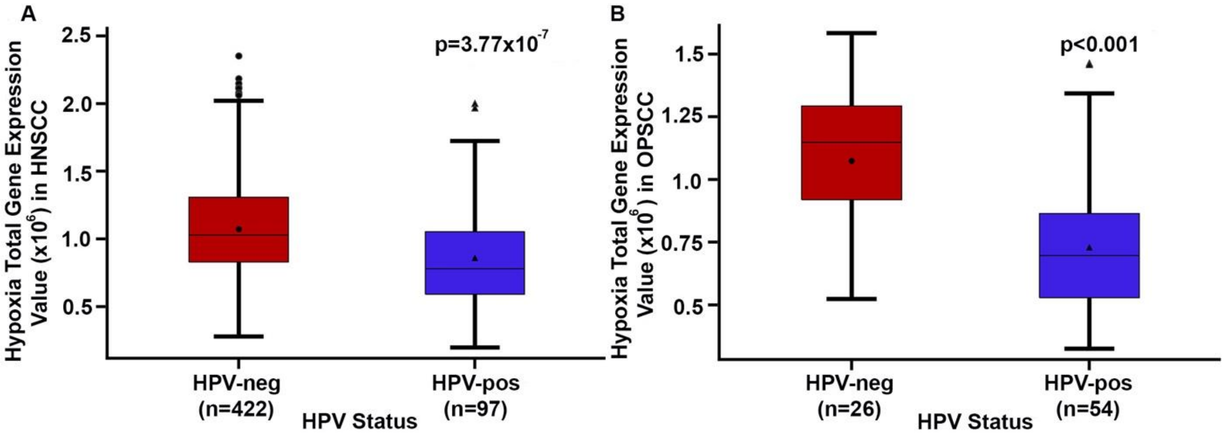

Supplementary Figure 5: Comparison of 26-gene hypoxia gene signature in HPV-pos versus HPV-neg HNSCC tumors. (A) Box whisker plot showing expression of the 26-gene hypoxia signature in HPV-neg (n=422) and HPV-pos (n=97) HNSCC patients ($p=3.77x10^{-7}$). **(B)** Box whisker plot showing expression of the 26-gene hypoxia signature in HPV-neg (n=26) and HPV-pos (n=54) OPSCC patients (p<0.001).

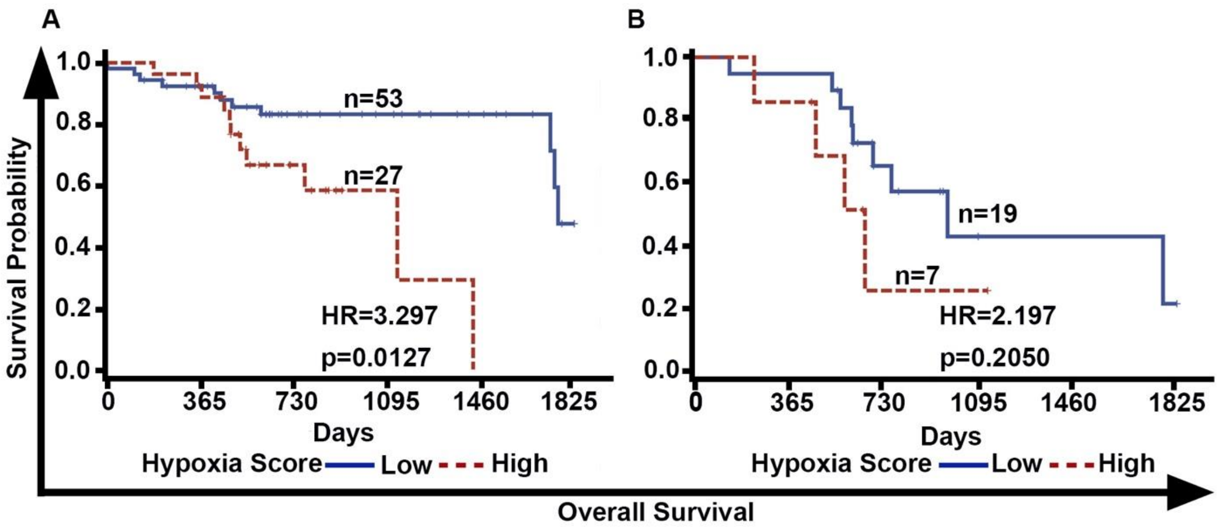

Supplementary Figure 6: (A) and (B) Kaplan Meier survival curves representing the survival probabilities of high and low hypoxia score OPSCC patients (HR=3.297; p=0.0127) and the survival probabilities of high and low hypoxia score hypoxia HPV-neg OPSCC patients (HR=2.197; p=0.2050), respectively.

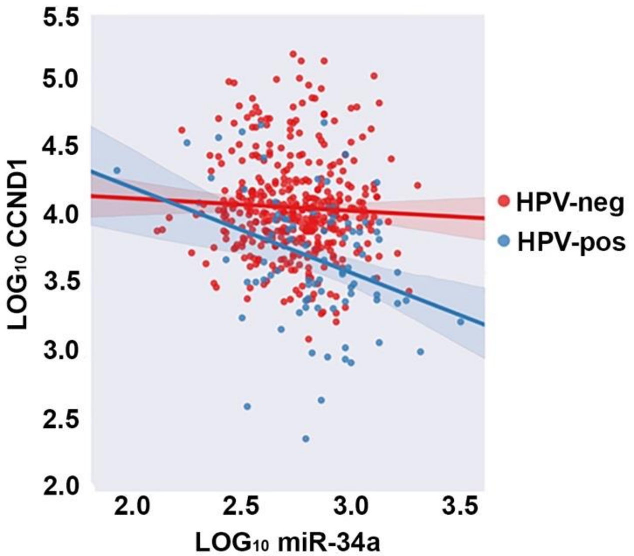

Supplementary Figure 7: *CCND1* gene expression is negatively correlated with the miR-34a expression in HPV-pos HNSCCs. Scatter plot representing the correlation between *CCND1* gene expression and miR-34a expression in HPV-pos and HPV-neg HNSCCs. The red line indicates the correlation for HPV-neg and blue line indicates correlation for HPV-pos.

Baseline Characteristics	HPV-neg	HPV-pos	p value
**Gender, n (%)**			
Female	129 (30.42)	12 (12.37)	0.0003
Male	295 (69.58)	85 (87.63)	
**Tumor Grade, n (%)**			
1	61 (14.39)	2 (2.06)	<0.0001
2	261 (61.56)	46 (47.42)	
3	91 (21.46)	31 (31.96)	
4	1 (0.24)	6 (6.19)	
X	8 (1.88)	10 (10.31)	
N/A	2 (0.47)	2 (2.06)	
**Tumor Stage, n (%)**			
I	16 (3.77)	5 (5.15)	0.0859
II	85 (20.04)	12 (12.37)	
III	89 (21.19)	16 (16.50)	
IVa	206 (48.58)	60 (61.86)	
IVb	8 (1.88)	3 (3.09)	
IVc	6 (1.42)	1 (1.03)	
N/A	14 (3.30)	0 (0.00)	
**Tumor Site, n (%)**			
Alveolar	13 (3.06)	5 (5.15)	<0.0001
Base of the tongue	12 (2.83)	16 (16.49)	
Buccal mucosa	23 (5.42)	0 (0.00)	
Floor of mouth	57 (13.44)	4 (4.12)	
Hypopharynx	5 (1.18)	5 (5.15)	
Larynx, nos	107 (25.24)	5 (5.15)	
Lip	3 (0.70)	0 (0.00)	
Oral cavity, nos	65 (15.33)	7 (7.22)	
Oral tongue	68 (16.03)	12 (12.37)	
Oropharynx	7 (1.65)	2 (2.06)	
Palate, hard	6 (1.42)	1 (1.03)	
Tongue, nos	48 (11.32)	2 (2.06)	
Tonsil	7 (1.65)	38 (39.18)	
N/A	3 (0.70)	0 (0.00)	
**Smoking, n (%)**			
1	92 (21.69)	30 (30.92)	0.1826
2	153 (36.08)	23 (23.71)	
3	59 (13.91)	13 (13.40)	
4	106 (25.00)	30 (30.92)	
5	2 (0.47)	0 (0.00)	
N/A	12 (2.83)	1 (1.03)	

Supplementary Table 1. Descriptive statistics of clinicopathological characteristics for HNSCC patients in the cohort (TCGA) used for *in silico* analysis of the prognostic value of CA7 signature.

	Normalized HPV-pos	Normalized HPV-neg	Fold Change (FC)	p-value
hsa-miR-490	1.353	0.225	6.0258	0.300
hsa-miR-449	15.589	4.767	3.270	0.204
hsa-miR-124	0.416	0.150	2.773	0.335
hsa-miR-30a	75518.415	52389.493	1.441	0.281
hsa-miR-24	231.0938	169.0763	1.367	0.000117
hsa-miR-34a	782.462	591.0943	1.324	0.000248
hsa-miR-769	164.319	127.123	1.293	$7.67x10^{-7}$
hsa-miR-32	225.810	182.773	1.235	0.000586
hsa-miR-128	736.136	623.156	1.181	0.0615
hsa-miR-195	158.353	154.902	1.022	0.860
hsa-miR-182	107714.647	107089.437	1.006	0.970
hsa-miR-339	259.434	258.0265	1.005	0.933
hsa-miR-140	5075.168	5181.0928	0.980	0.753
hsa-miR-218	184.885	195.486	0.946	0.668
hsa-miR-296	21.716	27.538	0.789	0.111
hsa-miR-144	754.152	969.679	0.778	0.0764
hsa-miR-409	125.684	162.474	0.774	0.00348
hsa-miR-1827	0.00560	0.00924	0.606	0.689
hsa-miR-520a	3.568	23.456	0.152	0.298

Supplementary Table 2. List of CA associated miRNAs sorted by logFC values. FC values represent a ratio of HPV-pos and HPV-neg values.

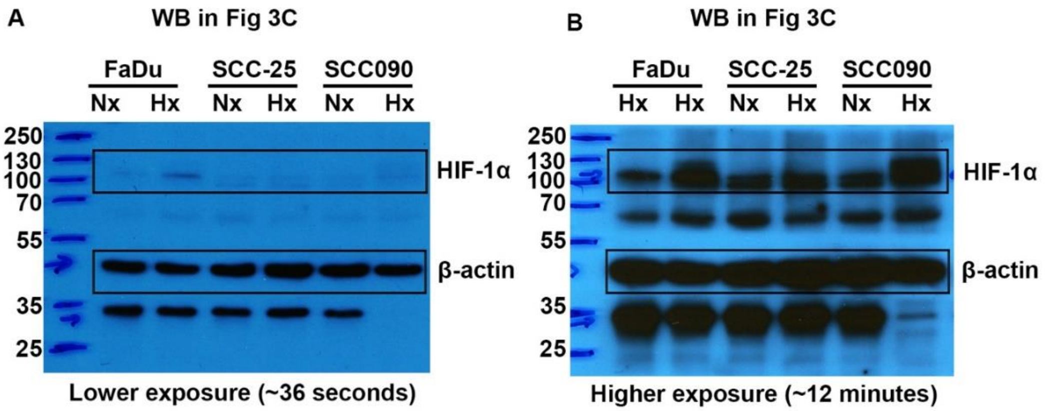

Supplementary Figure 8: Whole blot showing all the bands with all molecular weight markers are shown for Figure 3C.

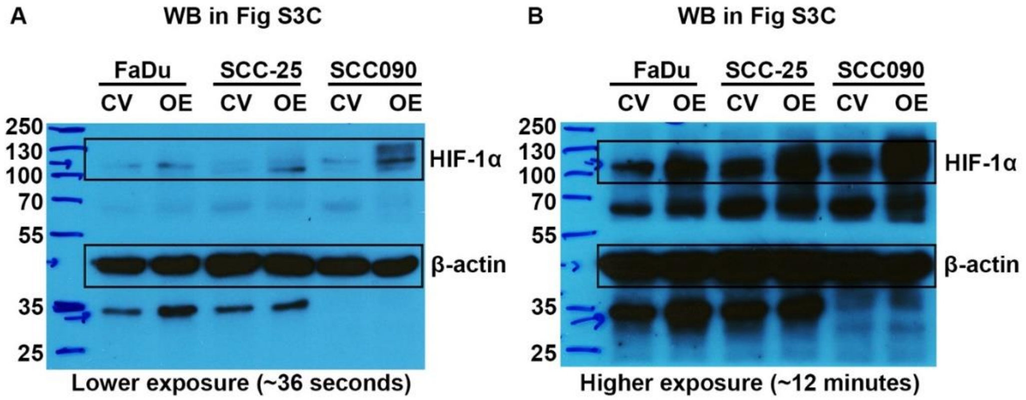

Supplementary Figure 9: Whole blot showing all the bands with all molecular weight markers are shown for Supplementary Figure 3C.

Experiment	Cells and condition	HIF-1α
Figure 3C Hypoxia Treatment	FaDu Nx	0.569983
	FaDu HX	1.089567
	SCC-25 Nx	0.768536
	SCC-25 Hx	1.131655
	SSC090 Nx	0.720258
	SSC090 Hx	1.215652
Supplementary Figure 3 C HIF-1α OE	FaDu CV	0.82915
	FaDu OE	1.64093
	SCC-25 CV	0.87774
	SCC-25 OE	1.95665
	SSC090 CV	0.85172
	SSC090 OE	4.044744

Supplementary Table 3: Densitometry values relative to loading control β-actin calculated using Image-J for immunoblot assays provided in main manuscript and supplementary data.
